# *Amomum tsaoko* flavonoids attenuate ulcerative colitis by inhibiting TLR4/NF-κB/NLRP3 signaling pathway and modulating gut microbiota in mice

**DOI:** 10.3389/fmicb.2025.1557778

**Published:** 2025-03-10

**Authors:** Zelin Huang, Yan Zhao, Weixing Yang, Chunfen Duan, Jun Sheng, Yang Tian, Lei Peng, Xiaoyu Gao

**Affiliations:** ^1^Yunnan Key Laboratory of Precision Nutrition and Personalized Food Manufacturing, Yunnan Agricultural University, Kunming, China; ^2^College of Food Science and Technology, Yunnan Agricultural University, Kunming, China; ^3^Engineering Research Center of Development and Utilization of Food and Drug Homologous Resources, Ministry of Education, Yunnan Agricultural University, Kunming, China; ^4^Division of Science and Technology, Yunnan Agricultural University, Kunming, China

**Keywords:** *Amomum tsaoko*, flavonoids, ulcerative colitis, *Escherichia-Shigella*, *Akkermansia*, *Bifidobacterium*, *Colidextribacter*, *Oscillibacter*

## Abstract

Ulcerative colitis (UC) is a global disease for which there is little of effective treatment options, thus creating an urgent need for the development of new drug candidates from natural and functional foods. *Amomum tsaoko* Crevost et Lemarie is a kind of medicinal and food herb that is rich in flavonoids. However, the pharmacological effects of *Amomum tsaoko* flavonoids (ATF) on UC have not yet been reported. The present study established a mouse model of UC using 3% dextran sulfate sodium (DSS), and modern molecular biology techniques such as IHC, RT-qPCR, Western blot, and 16S rRNA gene analysis were used to investigate the effect of ATF in UC mice. The results demonstrated that a high dose of ATF (100 mg/kg) led to a significant restoration of body weight, disease activity index score, and colon length, in addition to ameliorating colonic tissue damage in UC mice. ATF reduced the serum levels of lipopolysaccharides (LPS), inhibited the activation of the colonic TLR4/NF-κB/NLRP3 signaling pathway, and increased the mRNA expression of tight junction proteins such as *ZO-1*, *Occludin*, and *Claudin4*. Furthermore, ATF was found to reduce the relative abundance of the DSS-induced conditional pathogenic (*Escherichia-Shigella*, *Colidextribacter*, and *Oscillibacter*), increase the potential probiotic taxa (*Akkermansia*, *Bifidobacterium* and *unclassified_f__Atopobiaceae*). Interestingly, these genera were found to be significantly correlated with the UC core phenotypic indicators. These findings indicated that ATF may alleviate UC symptoms by modulating the gut microbiota-LPS/TLR4/NF-κB/NLRP3 axis. The present study has the potential to serve as a valuable reference point for understanding the role of natural flavonoids in the prevention of inflammatory diseases, and to expand the future applications of ATF in the fields of food and medicine.

## 1 Introduction

Ulcerative colitis (UC) is a chronic inflammatory bowel disease (IBD) that is characterized by periodic relapses, most often with recurrent abdominal pain and diarrhea, mucus-thickened blood stools, severe weight loss, and a long-term inflammatory state of the colon. These clinical manifestations increase the risk of colon cancer (Sun et al., 2021). The aetiology of UC is multifaceted and closely associated with immune dysfunction (Powell et al., 2015), impaired intestinal barrier function (Zhen and Zhang, 2019) and dysbiosis of the gut microbiota (Kim S. G. et al., 2019). At this juncture, the management of UC remains largely dependent on the utilization of pharmacological agents, predominantly anti-inflammatory drugs, immunosuppressants and biosuppression, such as sulfasalazine, azathioprine and mesalazine. However, these therapeutic modalities have been associated with an increased risk of disease exacerbation and deterioration (Klein and Eliakim et al., 2010; Pauly et al., 2017). It is therefore imperative to identify a safe and effective natural edible resource as an alternative drug for the treatment of UC.

In China, herbal medicine represents a typical and long-established intervention for the treatment of intestinal inflammation, utilizing plant-derived active ingredients. The composition of Chinese herbal medicine is complex, comprising large molecules that are primarily polysaccharides and water-soluble proteins, while small molecules may consist of flavonoids, alkaloids, amino acids, organic acids, nucleotides, and other substances. These small molecules generally exhibit fewer side effects. It has been established that the anti-UC properties of active compounds derived from herbal medicines primarily target inflammation or oxidative stress, which is associated with increased levels of anti-inflammatory cytokines (IL-4, IL-10, SOD), and decreased levels of pro-inflammatory cytokines (TNF-α, IL-1β, and IL-6 etc.) (Cao et al., 2019). These findings suggest that the study of natural drug compounds related to UC is of great value.

A substantial body of evidence from previous studies has demonstrated that flavonoids can alleviate IBD through a range of mechanisms. To illustrate, soybean isoflavones have been demonstrated to impede IκB protein phosphorylation and IKK degradation (Kim S. E. et al., 2019). Naringenin inhibits the TLR4/NF-κB pathway, inducing M2 macrophages and accelerating colonic recovery (Chaen et al., 2019). Quercetin enhances intestinal barrier function through the assembly of zonula occludens-2, occludin, and claudin-1 and the expression of claudin-4 (Suzuki and Hara, 2009). Nobiletin targets oxidative stress, NLRP3 inflammatory vesicle activation and immune responses in UC mice (Yang et al., 2024). Kaempferol alleviates colitis by restoring the gut microbiota and inhibiting the LPS-TLR4-NF-κB axis (Qu et al., 2021). The overall pharmacological mechanisms by which flavonoids alleviate UC appear to be related to the modulation of the body’s immune response, inflammatory response, intestinal barrier and gut microbiota.

*Amomum tsaoko* Crevost et Lemarie (AT) is one of the most widely used Chinese medicinal herbs, with a multitude of applications. It is also used as a spice to remove the fishy flavor and has the effect of warming the spleen and stomach, stopping vomiting, eliminating the hostage, and expelling the cold and pain in the abdomen. Nevertheless, AT is predominantly employed as a seasoning, and its potential therapeutic benefits remain largely untapped (Hu et al., 2018). The gradual study of AT has resulted in the isolation of over 300 compounds, with flavonoids representing the primary components of AT (Yang S. et al., 2022; Cai et al., 2021). The flavonoids present in AT have been demonstrated to possess high antioxidant activity, which has been shown to significantly improve the symptoms of type II diabetic mice (Zhang et al., 2022). Moreover, these flavonoids have been demonstrated to inhibit the production of NO in LPS-stimulated RAW 264.7 murine macrophages, which have been shown to exhibit potent anti-inflammatory activity (Whan et al., 2018). The flavonoids have additionally been demonstrated to display enhanced pharmacological activity in mice with constipation (Hu et al., 2023). These previous studies implied that the flavonoids present in AT may possess ameliorative properties in the context of IBD. However, no studies have been reported on the utilization of total flavonoids in AT (ATF) in IBD. In the present study, the anti-UC properties of high-purity ATF, which had been successfully prepared in our previous work (Hu et al., 2018) were evaluated in the experimental UC mouse model induced by dextran sulfate sodium (DSS). Furthermore, the anti-UC mechanism of ATF was explored from the perspectives of inflammatory signaling pathway and intestinal microecology, with a view to providing theoretical bases for the application of ATF in functional foods or medicines.

## 2 Materials and methods

### 2.1 Preparation of ATF

The AT fruits were sourced from Gongshan County, Yunnan Province of China. The preparation of ATF was outlined in brief, with reference to the published literature of our group (Huang et al., 2025). The AT was pulverized and then extracted using a sonicator. Subsequently, a specific concentration and pH of the upper sample solution were prepared. Adsorption and desorption were conducted with HPD300 macroporous resin, and the 20 and 30% ethanol eluates were concentrated and vacuum-dried, and stored for spare parts. The ATF obtained from the preparation primarily comprised (+)-Epicatechin (30.88%), Isoquercitrin (17.33%), Astragalin (9.52%) Kaempferol-3-O-rutinoside (8.46%), Procyanidin B2 (6.82%), and other compounds.

### 2.2 Design of animal experiments

A total of 60 male C57/6 J mice (20–23 g) were obtained from Beijing Vital River Laboratory Animal Technology Co., Ltd. (SPF grade). The animals were housed in the Yunnan key laboratory of precision nutrition and personalized food manufacturing. All the procedures were reviewed and approved by the life science ethics committee of Yunnan agricultural university (Approval no. 202312008). The animals were maintained at a temperature of 24 ± 1°C under a 12-h light/dark cycle, on an ad libitum diet (LAD3001M, Trophic Animal Feed High-tech Co. Ltd., China.) and water, and experiments were conducted in groups after 1 week acclimatization. The average body weight of mice in each group was similar.

**Figure 1:**
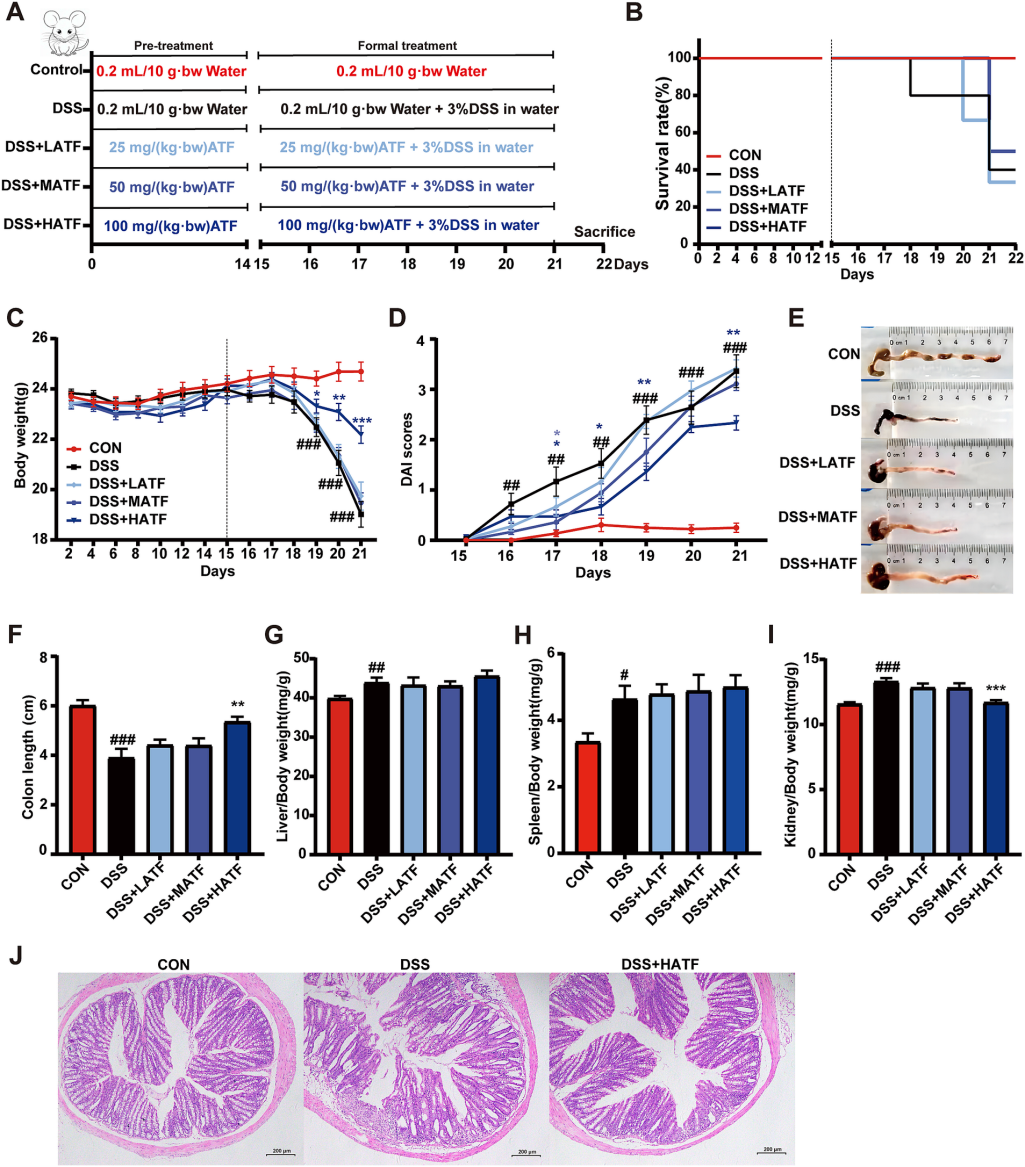
*Amomum tsaoko* flavonoids (ATF) is effective in alleviating symptoms of the DSS-induced colitis. **(A)** Animal experiment design; **(B)** survival curves; **(C)** mice body weights were recorded daily; **(D)** calculate the DAI score; **(E,F)** bowel images and colon length stats; **(G–I)** liver, spleen and kidney index; **(J)** images of colon sections stained with H&E. All data presented in mean ± SEMs, *n* = 10–12. # compared with the CON group; * compared with the DSS group. ^#^*p* < 0.05, ^##^*p* < 0.01, ^###^*p* < 0.001; *p < 0.05, **p < 0.01, ***p < 0.001.

Dextran sulfate sodium (DSS) is a common inducer of UC, and an acute UC model can usually be established by feeding with 3% DSS for 5–7 days. Five experimental groups were set up ad follows. (a) CON group (sterilized water, *n* = 12); (b) DSS group (3% DSS, *n* = 12); (c) DSS + LATF group (25 mg/kg·bw ATF + 3% DSS, *n* = 12); (d) DSS + MATF group (50 mg/kg·bw ATF + 3% DSS, *n* = 12); (e) DSS + HATF group (100 mg/kg·bw ATF + 3% DSS, *n* = 12). Gavage volume was set at 0.2 mL/10 g body weight. Figure 1A shows the experimental procedure. Fourteen days prior to the DSS intervention, mice were gavaged with sterilized water in the CON and DSS groups, and mice were gavaged with 25, 50, and 100 mg/kg-bw oxalic acid flavonoids in the ATF dose group, respectively. Water was freely consumed by the mice for the rest of the time. The 3% DSS solution was provided from day 15 to 22 to all groups except the CON. On the 21st day of the experiment, it was observed that the weight loss rate of mice in the model group exceeded 20%, which was considered to be the weight loss criterion for animal ethics. The experiment was therefore terminated on the 22nd day, and the mice were euthanized. Mice were executed with CO_2_ at the end day of experiment. Blood was collected and incubated to obtain serum. The livers, spleens and kidneys of the mice were weighed. The entire intestine was extracted, the mesentery and other associated tissues were excluded, and the proximal colon and cecum were harvested. The length of the colonic segments from each mouse was meticulously recorded. The colonic segments and the contents of the cecum were then collected and stored in a −80°C refrigerator for future use.

### 2.3 DAI score

During the course of DSS-induced UC, a daily record was kept of body weight change, fecal status and blood in stool in mice. The mean of the sum of the three scores was then used to calculate the disease activity index score (DAI). The calculation of weight loss was performed as the ratio of daily body weight change to initial body weight. The fecal status was slightly modified with reference to the Bristol stool typing method, and blood was discriminated by the instructions of the fecal occult blood kit (BC8270, Solarbio, Beijing). The detailed DAI scoring criteria are shown in Supplementary Table S1.

### 2.4 Serum biochemical analysis

Lipopolysaccharides, TNF-*α* and IL-1β in serum were detected by Mouse Lipopolysaccharides (LPS) ELISA Kit (CSB-E13066m, Cusabio, Wuhan), Mouse TNF-α ELISA Kit (E-EL-M3063, Elabscience, Wuhan) and Mouse IL-1β ELISA Kit (E-EL-M0037, Elabscience, Wuhan) were used for the assay. All the assays were performed according to the instructions of the kits.

### 2.5 RNA extraction, reverse transcription and real-time quantitative PCR

RNA was extracted from 10 to 20 mg of each mouse colon tissue sample using a bead-beating method and the FastPure Cell/Tissue DNA Isolation Mini Kit (RC101-01, Vazyme, Nanjing). RNA concentration and purity were determined using a spectrophotometer. Total RNA were then reverse transcribed to cDNA using HiScript III RT SuperMix for qPCR (+gDNA wiper) kit (R323-01, Vazyme, Nanjing). The obtained cDNA was stored at −30°C.

The real-time quantitative PCR reactions system was prepared according to the instructions of the ChamQ Universal SYBR qPCR Master Mix kit (Q711-02, Vazyme, Nanjing). Gene expression was determined using a LightCycler^®^ 480 System (Roche Diagnostics, Switzerland) PCR instrument, as outlined in the Supplementary Table S2. PCR reactions were set up in duplicate. As the reference gene, RPL-19 was used to calibrate target mRNA expression. The 2^-ΔΔCt^ method was used to calculate the results, with primer sequences given in Supplementary Table S3.

### 2.6 H&E staining of colonic tissue

A 4% formaldehyde solution was used to fix the colon. After embedding, all the colons were then cut into 3 μm sections for hematoxylin and eosin (H&E) staining. Two H&E-stained sections were obtained from each mouse for analysis. H&E-stained sections were imaged using a biological microscope.

### 2.7 Immunohistochemistry

The 3 μm wax blocks were dewaxed in xylene and rehydrated in graded ethanol. Following quenching of endogenous peroxidase activity and blocking of non-specific binding, the sections were subjected to an overnight incubation at room temperature with F4/80, TLR4, and NLRP3 mouse monoclonal antibodies (28463-1-AP, 66350-1-Ig, and 68,102-1-Ig respectively, Proteintech, Wuhan). This was followed by an incubation for 20 min at room temperature with biotinylated secondary antibodies (Zymed Laboratories, Carlsbad, CA, United States). The slides were then incubated with reagents from the Avidin-Biotin (SP-2001, Vector Laboratories, CA, United States) and 3,3′-diaminobenzidine kits (SK-4100, Vector Laboratories, CA, United States). Images obtained using Olympus CX43 microscope and CellSens Entry. The sections were photographed top-to-bottom and left-to-right and images of undamaged tissue structure were selected for analysis. The images were digitized using Image Pro-Plus 6.0 software (Media cybernetics, United States).

### 2.8 16S rRNA gene sequencing

Samples of cecal content were sent to Shanghai Majorbio Bio-pharm Technology Co., Ltd. for 16S rRNA gene sequencing. The microbial DNA was extracted from the cecum contents of the mice in the CON, DSS and DSS + HATF groups using the E.Z.N.A.^®^ Soil DNA kit (Omega Bio-tek, United States). Final DNA concentration and purity were determined using a NanoDrop 2000 UV–Vis (Thermo Scientific, United States). The DNA quality was evaluated using 1% agarose gel electrophoresis. The V3–V4 highly variable region of the bacterial 16S rRNA gene was amplified using a thermal cycling PCR system (GeneAmp 9,700, ABI, United States) and primers 338F (5′- ACTCCTACGGGGAGGCAGCAG3’) and 806R (5’- GGACTACHVGGGTWTCTAAT -3′). PCR was performed using the following procedure: denaturation at 95°C for 3 min; annealed at 95°C and 55°C for 30 s, respectively, and extended at 72°C for 45 s, 27 cycles; extended at 72°C for 10 min. PCR was conducted three times in a 20 μL mixture comprising 4 μL of 5 × FastPFU buffer, 2 μL of 2.5 mM dNTPs, 0.8 μL of each primer (5 μM), 0.4 μL of FastPFU polymerase, and 10 ng of template DNA. PCR products obtained from 2% agarose gels were purified using the Axyprep DNA Gel Extraction Kit (Axygen Biosciences, United States) and quantified using Quantifluor™-ST (Promega, United States) according to the manufacturer’s protocol. The purified amplicons were pooled in equimolar amounts and subjected to paired-end sequencing (2 × 300) on the Illumina MiSeq platform (Illumina, United States). The RDP Classifier was used to classify and analyze the Silva (SSU123) 16S rRNA database. Bioinformatics analysis was conducted on Majorbio’s cloud platform.

### 2.9 Data analysis

Data analysis were conducted using GraphPad Prism 8.0, with results expressed as the means ± standard errors of the means (SEMs). Analysis of variance was conducted using the statistical software package SPSS 26.0 (SPSS Inc., Chicago, IL, United States). Statistical analysis of the results was conducted using analysis of variance (ANOVA) with Duncan’s test. Advanced Cor link was performed using the OmicStudio tools at https://www.omicstudio.cn/tool. Multivariate analyses were conducted using the Majorbio platform. Unless stated otherwise, results were statistically significant at *p* < 0.05.

## 3 Results

### 3.1 ATF relieved the DSS-induced colitis in mice

Figure 1A illustrates the flow of the animal experiment. The CON group showed no unusual symptoms during the experiment. The DSS group, the DSS + LATF group, the DSS + MATF group, and the DSS + HATF group, respectively, had mortality in 2, 2, 1, and 0 mice following DSS ingestion (Figure 1B). From day 5, DDS-treated mice lost weight (*p* < 0.01) and had diarrhea and blood in their stool. They showed a big increase in DAI score compared to the CON group (*p* < 0.01). The DSS + HATF group showed a strong effect on body weight and DAI score (*p* < 0.01, Figures 1C,D). Interestingly, low, medium, and high doses of ATF inhibited colon shortening and alleviated DSS-induced liver, spleen, and kidney swelling, with the high dose exhibiting a pronounced effect (*p* < 0.01, Figures 1E–I). Pathological sections showed severe damage to the mucosal layer of the colon in mice in the DSS group, with a marked reduction in crypt and goblet cells. The DSS + HATF group showed less inflammation than the model group, with more goblet cells and a more intact mucosal structure (Figure 1J). In sum, high doses of ATF could effectively treat UC in mice.

### 3.2 ATF could regulate the expression of inflammatory factors

**Figure 2:**
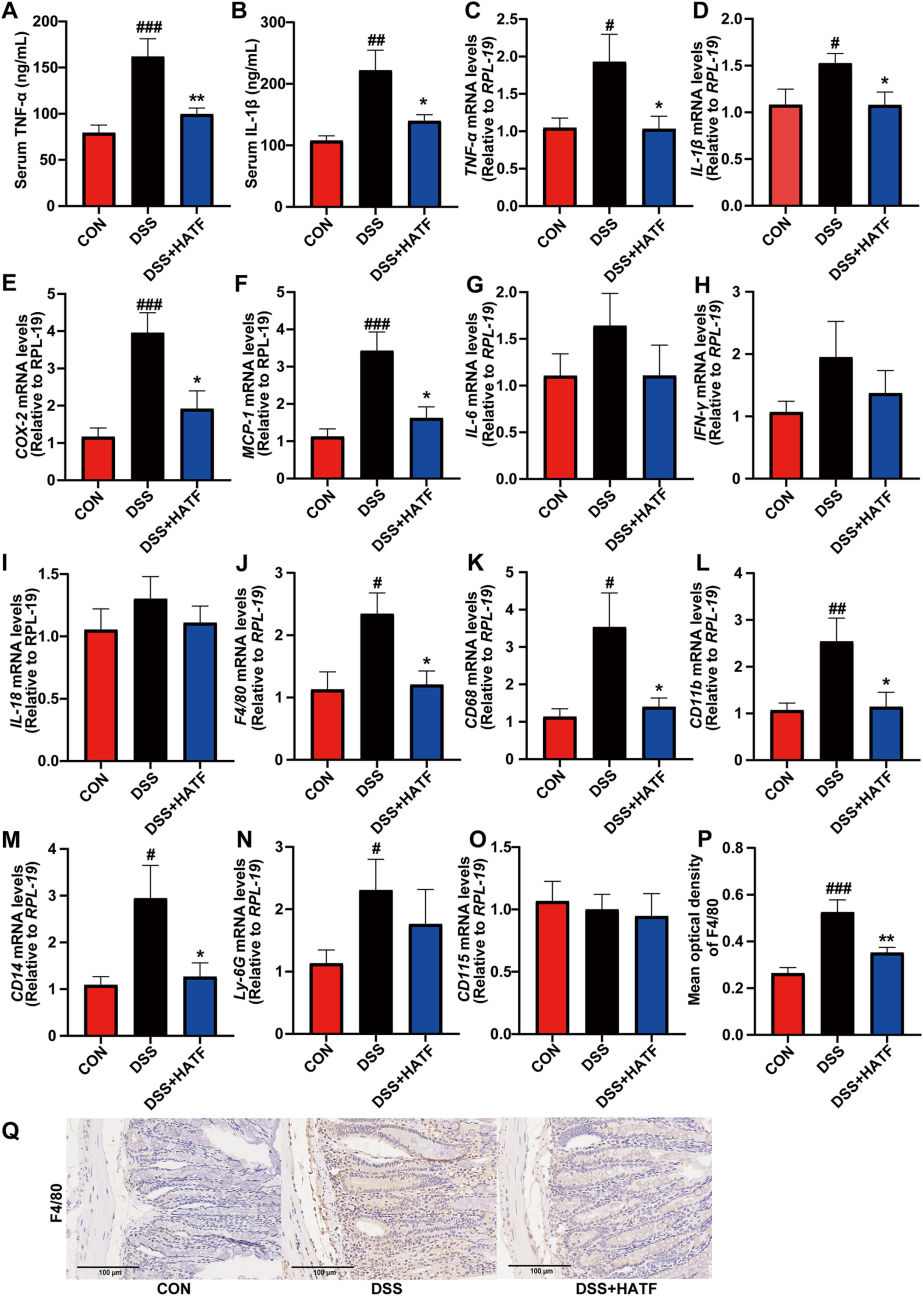
*Amomum tsaoko* flavonoids reversed the DSS-induced up-regulation of pro-inflammatory factors and inflammatory cell infiltration. **(A,B)** Serum levels of TNF-α and IL-1β; **(C–O)** the mRNA expression of *TNF-α*, *IL-1β*, *COX-2*, *MCP-1*, *IL-6*, *IFN-γ*, *IL-18*, *F4/80*, *CD68*, *CD11b*, *CD14*, *Ly-6G (Gr-1)*, and *CD115*. **(P,Q)** Percentage of F4/80 positive area in colonic tissue, and represented IHC images in three group. All data presented in mean ± SEMs, *n* = 8–10. # compared with the CON group; * compared with the DSS group. ^#^*p* < 0.05, ^##^*p* < 0.01, ^###^*p* < 0.001; *p < 0.05, **p < 0.01.

Abnormal increases in the levels of inflammatory factors are an important feature of UC. As shown in Figure 2, serum levels TNF-*α* and IL-1β, plus colonic *TNF-α*, *IL-1β*, *COX-2*, and *MCP-1* mRNA levels were higher in the DSS group than CON. The ATF intervention significantly reduced the levels of these factors (*p <* 0.05, Figures 2A–F) and tended to down-regulate *IL-6, IFN-γ*, and *IL-18* mRNA expression, though not statistically significant (*p* > 0.05, Figures 2G–I). The findings showed that the ATF decreased the DSS-induced inflammation in UC.

Similarly, ATF intervention reversed the DSS-induced up-regulation of the mRNA expression of colonic *F4/80, CD68, CD11b, CD14,* and *Ly-6G* (*p* < 0.05), with no significant effect on *CD115* (*p* > 0.05, Figures 2J–O). Furthermore, immunohistochemistry was utilized to investigate the expression of the macrophage marker, F4/80, in mice. The analysis revealed that F4/80 expression within the colon exhibited an increase in the DSS-induced UC model, concurrent with a decrease in response to ATF treatment (Figures 2P,Q). These observations are consistent with the results obtained from RT-qPCR, suggesting that ATF exerts a regulatory effect on inflammatory responses and contributes to the preservation of colonic tissue integrity.

### 3.3 ATF reduced the DSS-induced endotoxaemia and damage to the intestinal barrier

**Figure 3:**
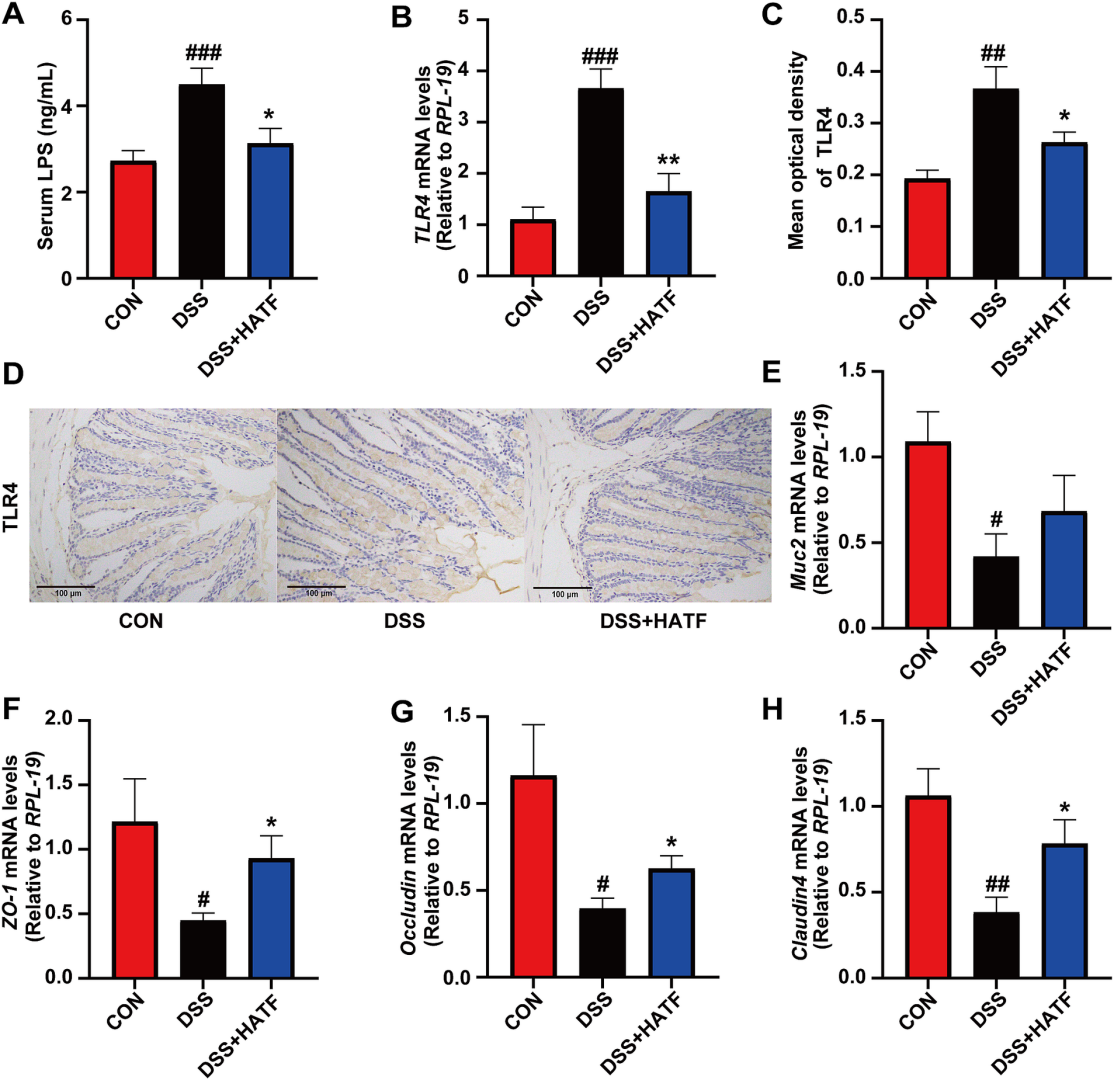
*Amomum tsaoko* flavonoids alleviated metabolic endotoxaemia and ameliorated intestinal barrier damage. **(A)** Serum LPS levels. **(B)** The mRNA expression of TLR4 in colon. **(C,D)** Percentage of TLR4 positive area in colon, and represented IHC images in three group. **(E–H)** The mRNA expression of Muc2, ZO-1, Occludin, and Claudin4, respectively. All data presented in mean ± SEMs, *n* = 8–10. # compared with the CON group; * compared with the DSS group. ^#^*p* < 0.05, ^##^*p* < 0.01, ^###^*p* < 0.001; *p < 0.05, **p < 0.01.

The release of toxins from the body is intimately associated with disrupting the gut barrier (Di Tommaso et al., 2021). Serum LPS and TLR4 levels in the colon increased in mice in the DSS group compared to the CON group (*p <* 0.01), indicating metabolic endotoxemia. LPS and TLR4 levels were lower in the ATF group than the DSS group (*p* < 0.05). TLR4 immunohistochemistry results were similar to the ATF group and close to those of the CON group (Figures 3A–D). In parallel, the mRNA expression of *Muc2, ZO-1, Occludin* and *Claudin4* were markedly reduced in the DSS-induced colitis mice (*p* < 0.05). Despite the absence of a notable impact of ATF on *Muc2* (*p* > 0.05, Figure 3E), it was observed to stimulate the mRNA expression of *ZO-1, Occludin,* and *Claudin4* (*p* < 0.05, Figures 3F–H). In general, the ATF could modulate these genes to match control levels, reducing metabolic endotoxaemia and intestinal damage.

### 3.4 ATF inhibited the NF-κB/NLRP3 inflammatory pathway

**Figure 4:**
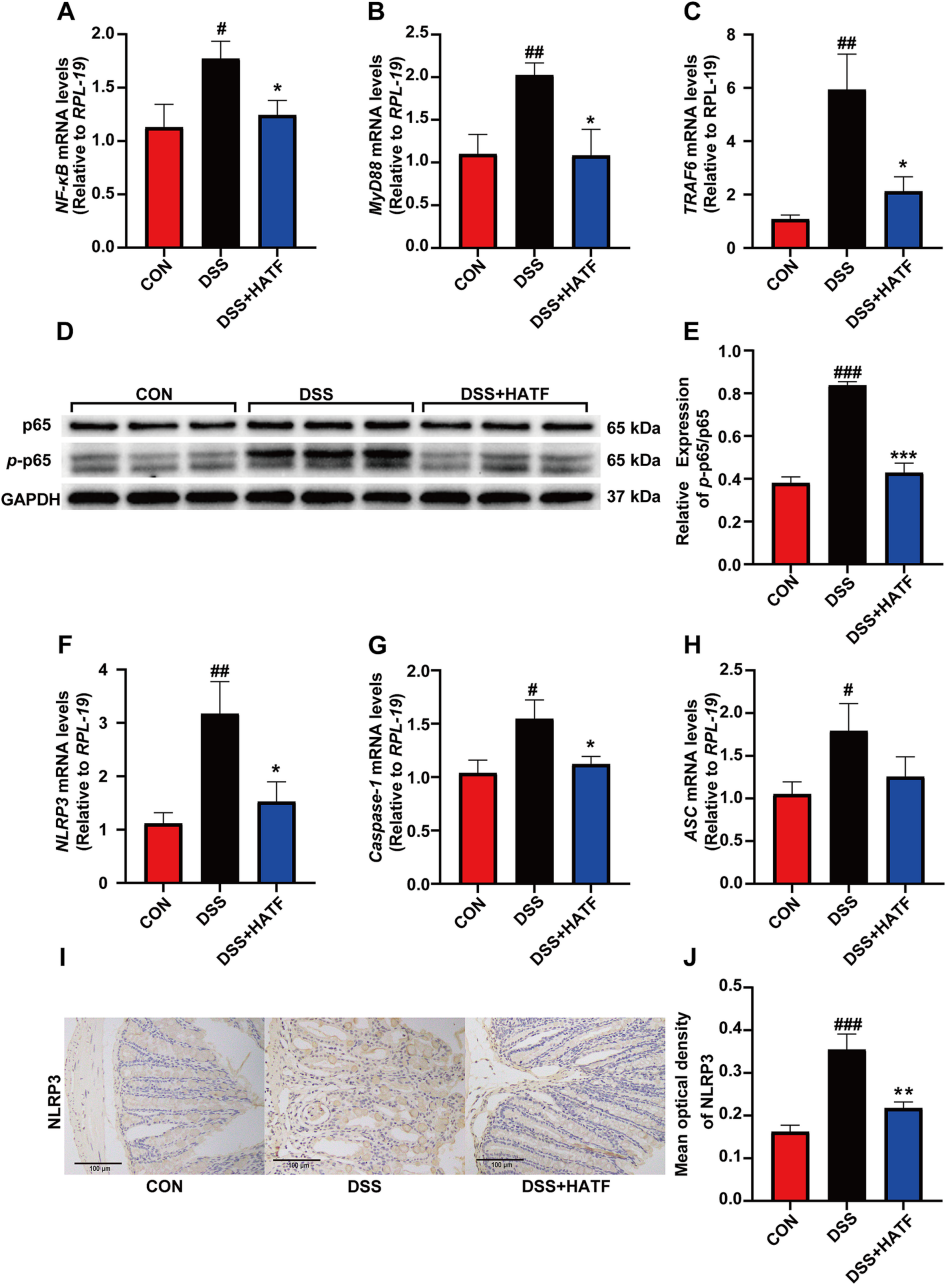
The effect of ATF on the NF-κB and NLRP3 signaling pathway. **(A–C)** The mRNA expression of *NF-κB*, *MyD88*, *TRAF6* in colon, respectively. **(D,E)** The phosphorylation of p65 was detected by Western blotting, and the relative expression of *p*-p65/p65. **(F–H)** The mRNA expression of *NLRP3*, *Caspase-1*, and *ASC* in colon, respectively. **(I,J)** Represented IHC images in three group, and percentage of NLRP3-positive area in colon. All data presented in mean ± SEMs, *n* = 8–10. # compared with the CON group; * compared with the DSS group. ^#^*p* < 0.05, ^##^*p* < 0.01, ^###^*p* < 0.001; *p < 0.05, **p < 0.01.

Given that the DSS contributed to the increased levels of LPS and TLR4, we postulated that the NF-κB/NLRP3 pathway was activated (Liu et al., 2023). In DSS-induced colitis, the mRNA expression of *NF-κB, MyD88*, and *TRAF6* was elevated (*p* < 0.05, Figures 4A–C), while the expression of *p*-p65 was significantly up-regulated in the colons of mice in the DSS group (*p* < 0.01, Figures 4D,E). This finding suggests that the colonic NF-κB signaling pathway was activated in our UC mice. Concurrently, the mRNA expression of colonic *NLRP3, Caspase-1,* and *ASC* was elevated in the DSS group of mice compared with the CON group (*p* < 0.05, Figures 4F–H). And IHC analysis revealed that the expression level of colonic NLRP3 in mice in the DSS group was significantly higher than that in the CON group (*p* < 0.01, Figures 4I,J). Conversely, ATF was able to inhibit the expression of these genes and proteins to varying degrees, approaching the levels observed in the CON group. Overall, ATF could inhibit the activation of the NF-κB/NLRP3 signaling pathway and to reduce the expression of colonic inflammatory mediators, thereby ameliorating DSS-induced colonic inflammation.

### 3.5 ATF ameliorated the DSS-induced gut microbiota dysbiosis

**Figure 5:**
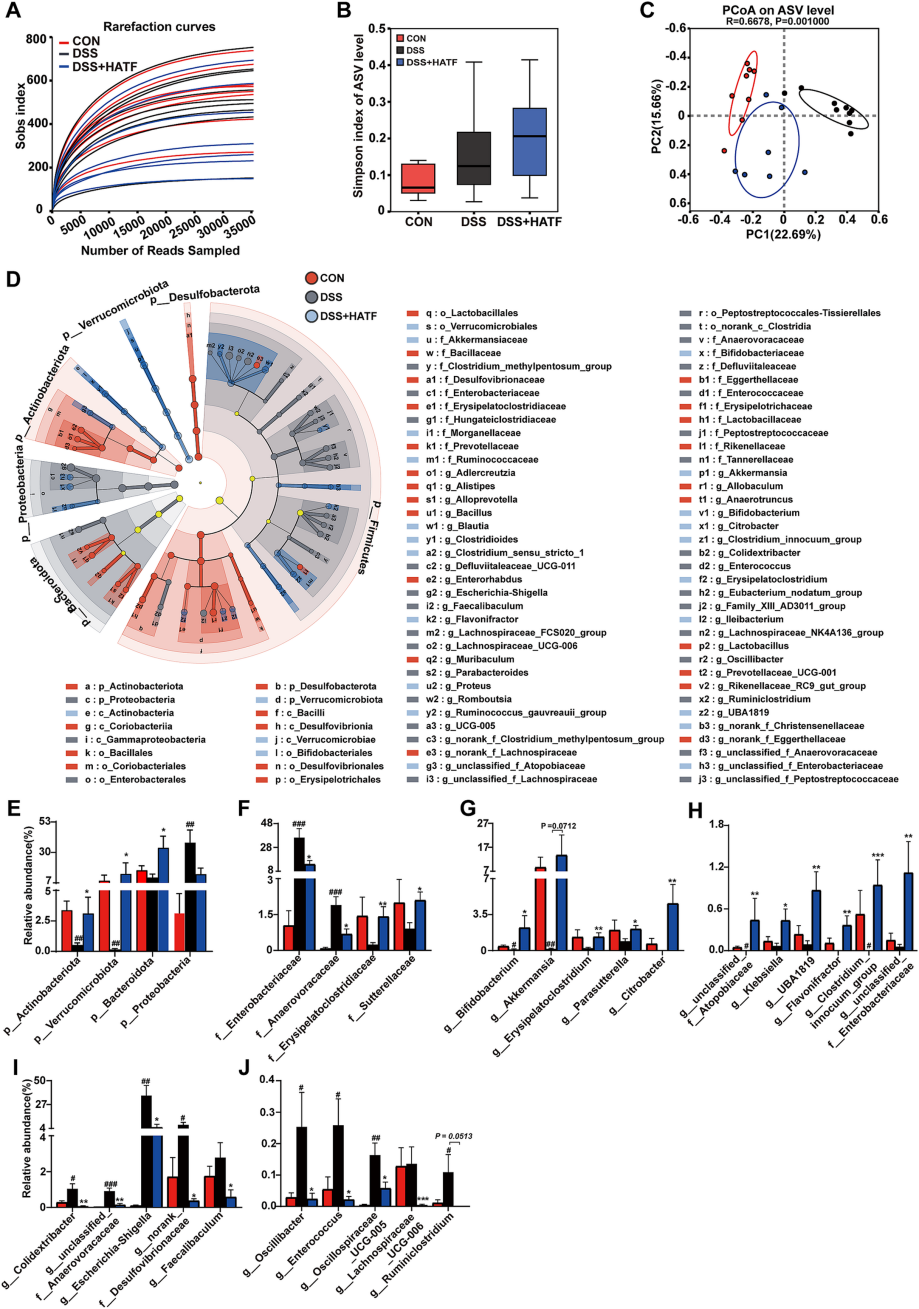
*Amomum tsaoko* flavonoids altered the structure and composition of the gut microbial community in UC mice. **(A)** Rank-abundance curve; **(B)** α-diversity index; **(C)** PCoA based on the Bray–Curtis distance algorithm. **(D)** Linear discriminant analysis effect size (LEfSe) analyses (LDA score > 2.0). **(E)** Relative abundance of microbes at the phylum level. **(F)** Relative abundances of microbes at the family level. **(G–J)** Relative abundances of microbes at the genus level. All data presented in mean ± SEMs, *n* = 6. # compared with the CON group; * compared with the DSS group. ^#^*p* < 0.05, ^##^*p* < 0.01, ^###^*p* < 0.001; *p < 0.05, **p < 0.01, ***p < 0.001.

The 16S rRNA gene was sequenced to assess the impact of ATF on the gut microbiota of the UC mice. The sparse curve of the Sobs index for each sample exhibited a tendency to flatten with an increase in the number of sequences, indicating that the sequencing depth was sufficient to reflect the diversity of the samples and that the sequencing results were credible (Figure 5A). Despite the DSS and DSS + HATF groups demonstrating no significant difference from the CON group with regard to *α*-diversity indices (Figure 5B), the PCoA result clearly separated the CON, DSS, and DSS + HATF group, with the DSS + HATF group demonstrating a closer proximity to the CON group (Figure 5C), suggesting that ATF altered the β-diversity of the gut microbiota in UC mice and had a restorative effect on the DSS-induced gut microbiota dysbiosis.

The LEfSe analyses similarly demonstrated the dramatic effects of loperamide and ATF on microbial taxa at different levels (Figure 5D). A total of 88 different taxa, including 4 phyla, 6 classes, 10 orders, 20 families and 48 genera, were identified in the three groups. Of these, the CON, DSS, and DSS + HATF groups had 31, 32, and 25 dominant taxa, respectively, (Figure 5D and Supplementary Figure S1). The present study focused on the taxa significantly affected by DSS and ATF, especially those significantly changed by ATF treatment.

At the phylum level, the relative abundance of *Actinobacteriota*, *Verrucomicrobiota,* and *Proteobacteria* in the cecum of mice in the DSS group was significantly different from that of the CON group (*p* < 0.01), and the ATF intervention significantly increased the relative abundance of *Actinobacteriota* and *Verrucomicrobiota* and *Bacteroidota* (*p* < 0.05), but did not restore Proteobacteria to a significant level (Figure 5E, *p* > 0.05). At the family level, DSS treatment induced a significant up-regulation of *Enterobacteriaceae* and *Anaerovoracaceae*, which was significantly reversed by ATF intervention (*p* < 0.05, Figure 5F). Furthermore, the relative abundance of *Erysipelatoclostridiaceae* and *Sutterellaceae* was induced to decrease by DSS, significantly elevated by ATF intervention, and significantly suppressed the increase in relative abundance of *Anaerovoracaceae* (*p* < 0.05, Figure 5F).

At the genus level, high doses of ATF elevated the relative abundance of *Bifidobacterium*, *Akkermansia*, *Erysipelatoclostridium*, *Parasutterella,* and *Citrobacter* to a greater extent and in a statistically significant way in comparison to the DSS group, as well as significantly increasing the number of relative abundance of *unclassified_f__Atopobiaceae*, *Klebsiella*, *UBA1819*, *Flavonifractor*, *Clostridium_innocuum_group* and *unclassified__f__Enterobacteriaceae* (*p* < 0.05, Figures 5G,H). In contrast, high doses of ATF significantly suppressed the abnormal increase in the relative abundance of *Colidextribacter*, *unclassified_f__Anaerovoracaceae*, *Escherichia-Shigella*, *norank_f__Desulfovibrionaceae*, *Faecalibaculum*, *Oscillibacter*, *Enterococcus*, *Oscillospiraceae_UCG-005*, *Lachnospiraceae_UCG-006*, and *Ruminiclostridium*. It is noteworthy that in DSS-induced UC mice, the relative abundance of colonies such as *Escherichia-Shigella*, *Desulfovibrionaceae*, and *Enterococcus*, which may produce LPS, was significantly increased, whereas ATF inhibited this change (*p* < 0.05, Figures 5I,J). These findings illustrate that ATF has the capacity to reinstate the DSS-induced gut microbial dysbiosis to varying extents across multiple domains, including microbial diversity, community structure, and species composition.

### 3.6 Correlation analysis between specific microbiological taxa and core indicators

In order to elucidate the pivotal function of gut microbiota in the alleviation of UC by ATF, bivariate correlation analyses were employed to investigate the correlations between microbial taxa that exhibited a significant response to ATF and core indicators in UC mice.

**Figure 6:**
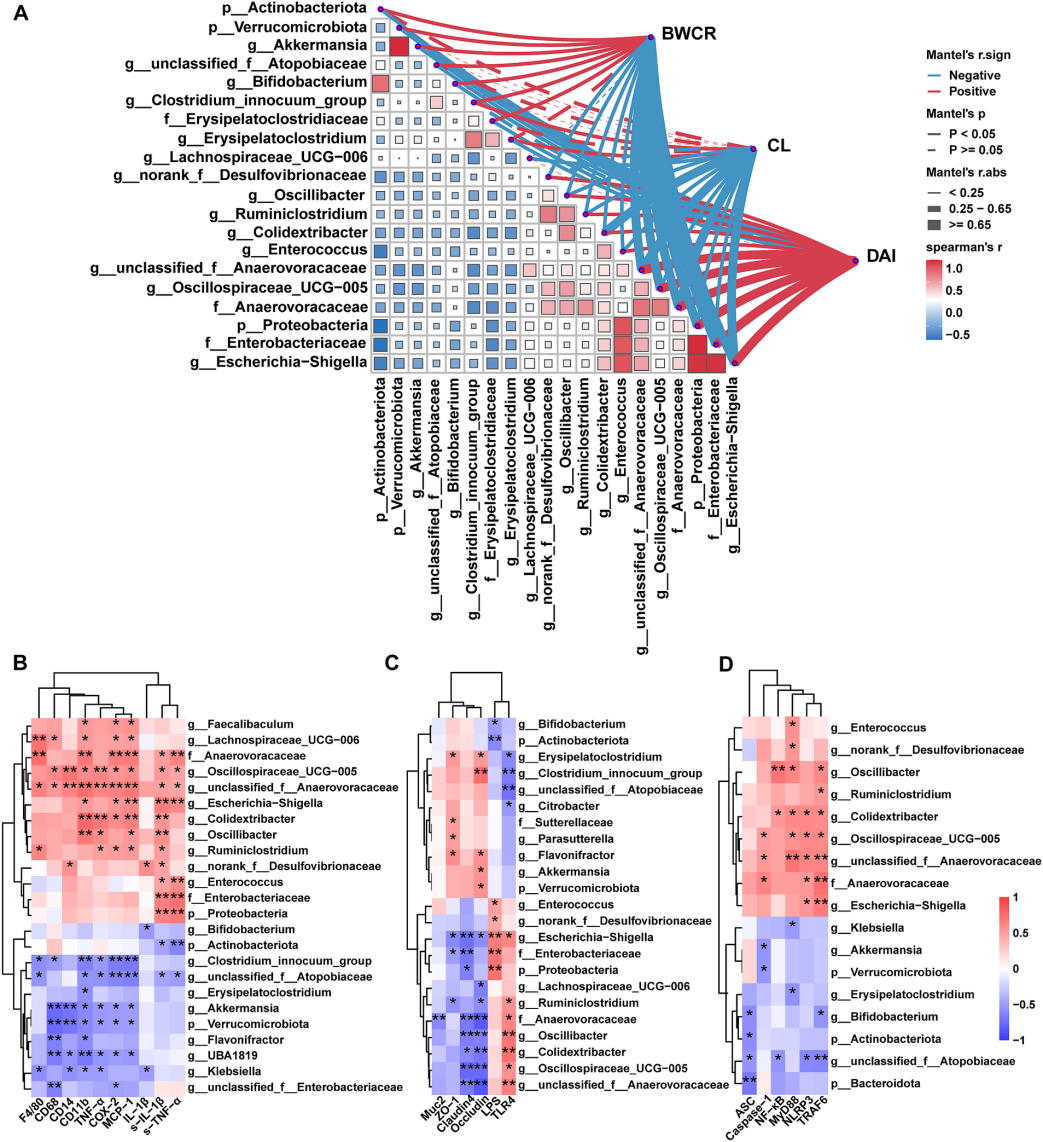
Correlations between the differential genera and core indicators of UC. **(A)** Correlations between differential genera and conventional pathological phenotypes of UC mice. **(B)** Correlations between differential genera and inflammatory factors and immune cell markers. **(C)** Correlations between differential genera and metabolic endotoxaemia and colonic barrier factors. **(D)** Correlations between differential genera and NF-κB/NLRP3 signaling pathway. BWCR, body weight change rate of mice; CL, colon length; DAI, disease activity index. S-IL-1β, IL-1β in serum; S-TNF-α, TNF-α in serum; LPS, LPS in serum; all other factors are relative expressions of mRNAs. The color at each point of intersection indicates the value of the *r* coefficient (*n* = 22–24). The *Benjamini–Hochberg* (BH) procedures were used to adjust *p* values for multiple testing. * indicates that there is a significant correlation between these two parameters (*p* < 0.05). *p < 0.05, **p < 0.01.

Figure 6A shows the link between microbial taxa and pathological phenotypes in UC mice. BWCR, CL and DAI are core parameters reflecting the extent of UC disease in mice. These parameters correlated with the differential microbial taxa obtained from the screening. All three parameters demonstrated a robust correlation with *Escherichia-Shigella*, *Anaerovoracaceae* and *unclassified_f__Anaerovoracaceae* (*r* ≥ 0.65 or *r* ≤ −0.65, *p <* 0.05). *Oscillospiraceae_UCG-005, Proteobacteria* and *Enterobacteriaceae* were negatively correlated with BWCR (*r* ≤ −0.65, *p <* 0.05) and positively correlated with DAI (*r* ≥ 0.65, *p <* 0.05). *Colidextribacter* and CL were negatively correlated (*r* = −0.6531, *p* < 0.05). There is a significant positive correlation between *Actinobacterota, Verrucomicrobiota, Akkermansia, unclassified_f__Atopobiaceae, Bifidobacterium*, *Erysipelatoclostridium* and BWCR (*r <* 0.65, *p <* 0.05). A significant negative correlation was evident between these variables and DAI (*r* > −0.65, *p <* 0.05). Correlations between *Oscillibacter, Ruminiclostridium*, and *Enterococcus* and BWCR and CL were negative (*r* > −0.65, *p* < 0.05), while those with DAI were positive. In general, BWCR and CL showed the opposite correlation observed in DAI results, consistent with conventional pathological UC signs in mice. Notably, the microbial taxa positively correlated with BWCR and CL were up-regulated by ATF. Those negatively correlated with BWCR and CL and positively correlated with DAI were increased by DSS. ATF significantly reversed this.

The relationship between ATF and other key indicators were also investigated. Our findings revealed a significant positive correlation between *Escherichia-Shigella, Anaerovoracaceae,* and *unclassified_f__Anaerovoracaceae* and serum TNF-*α*, IL-1β, and colonic *CD11b* and *TLR4*. These taxa were positively correlated with colonic *TRAF6* and *NLRP3* and negatively correlated with *Occludin* and *Claudin4* (Figures 6B–D). A significant positive correlation was observed between *unclassified_f__Anaerovoracaceae* and colonic *F4/80, CD68, CD14, TNF-α, COX-2, MCP-1, MyD88,* and *Caspase-1* (*p* < 0.05). A notable inverse correlation was observed between *unclassified_f__Atopobiaceae* and serum IL-1β, colonic *TNF-α*, *CD11b, TLR4* and *TRAF6*. Conversely, *Oscillibacter, Colidextribacter and Oscillospiraceae_UCG-005* had a different correlation, with a stronger positive association. There was also a significant negative correlation with *occludin* and *claudin4* (*p* < 0.05). In addition, *Escherichia-Shigella*, *norank_f__Desulfovibrionaceae*, *Enterococcus*, *Proteobacteria*, and *Enterobacteriacea*e showed a positive correlation with LPS, while *Actinobacteriota* and *Bifidobacterium* demonstrated a negative correlation. Remarkably, only *Escherichia-Shigella* was significantly positively correlated with both LPS and TLR4 (*p* < 0.05).

In conclusion, *Escherichia-Shigella, unclassified_f__Anaerovoracaceae*, *unclassified_f__Atopobiaceae*, *Bifidobacterium, norank_f__Desulfovibrionaceae*, *Colidextribacter,* and *Oscillibacter* correlated with multiple host core indicators, suggesting that gut microbiota may play a key role in the improvement of UC by ATF.

## 4 Discussion

Ulcerative colitis is associated with autoimmune function abnormalities. The disease has a long-lasting impact on affected individuals, limiting their quality of life. At the present time, DSS is currently widely used in UC modeling. DSS directly stimulates intestinal cells and disrupts the intestinal barrier, causing acute enteritis, which is the same as clinical UC presentation (Gadaleta et al., 2017). In the present study, DSS caused weight loss, bloody stools and shortened colons in mice. This shows that the DSS model is effective. It has been reported that the DSS-induced UC mice frequently exhibit an imbalance in intestinal homeostasis (Pei et al., 2019; Håkansson et al., 2015). Therefore, a systematic evaluation was conducted of the effects of ATF on the DSS-induced UC mice in terms of intestinal inflammation, intestinal barrier, and gut microbiota. Our results demonstrated that ATF intervention (100 mg/kg) markedly impeded the substantial decline in body weight, the surge in DAI scores, the reduction in colon length, and the disruption of colon tissue structure in UC mice. Additionally, the expression of imbalanced inflammatory factors was diminished, the compromised intestinal barrier was replenished, the activation of the LPS/TLR4/NF-κB/NLRP3 signaling pathway was suppressed, and the disrupted gut microbiota was ameliorated. These effects collectively resulted in the effective alleviation of UC symptoms in mice.

The therapeutic efficacy of ATF in UC may be due to its active ingredients. During the pre-trial period, a plant-wide targeted metabolomics assay was conducted, which revealed that the relative abundance of flavonoids in ATF was greater than 73%. The predominant flavonoids identified were (+)-Epicatechin (30.88%), Isoquercitrin (17.33%), Astragalin (9.52%) Kaempferol-3-O-rutinoside (8.46%), Procyanidin B2 (6.82%), and others. It was found that astragalin was capable of effectively modulating the relative abundance of *Ruminococcaceae* and *Escherichia-Shigella*, while concomitantly exerting a capacity to inhibit NF-κB activation in mice, thereby serving to attenuate DSS-induced acute experimental colitis (Peng et al., 2020). Kaempferol administration was found to exert a protective effect on UC mice by restoring the intestinal microflora and simultaneously inhibiting the LPS-TLR4-NF-κB axis (Qu et al., 2021). Proanthocyanidin extract was found to decrease the protein levels of NF-κB and Keap-1 and increase the expression of Nrf2 and HO-1 proteins in UC mice (Wang et al., 2022). Rutin was found to prevent inflammation by inhibiting the p38/MAPKAPK2 and PI3K/Akt/GSK3β/NF-κB signaling axes in UC mice (Sharma et al., 2021). It is noteworthy that astragalin, kaempferol, proanthocyanidin, and rutin all demonstrated the capacity to impede the activation of the NF-κB-related pathway. These monomers were identified as predominant constituents within the ATF, thereby offering substantial corroborating evidence for the findings of this study.

In the DSS-induced mouse model of colitis, it is frequently observed to occur concurrently with intestinal inflammation. Macrophage activation permits pro-inflammatory factors to assume an active role in the inflammatory process. Conversely, an excess of pro-inflammatory cytokines induces macrophage polarization, thereby intensifying the inflammatory response (Shapouri-Moghaddam et al., 2018). The present study observed that DSS stimulation increased inflammatory cell infiltration in colon of UC mice, significantly up-regulating the expression levels of inflammatory cell markers *F4/80, CD68, CD11b, CD14,* and *Ly-6G,* as well as the significant expression of the inflammatory factors *TNF-α, IL-1β, COX-2,* and *MCP-1*. This indicates that the ingestion of DSS may have triggered the secretion of pro-inflammatory factors by particular immune cells, thereby contributing to the onset of colitis. Nevertheless, flavonoids have the capacity to regulate the balance of the immune system directly. It has been demonstrated that flavonoid molecules can facilitate the conversion of macrophages from the M1 to the M2 phenotype, thereby alleviating colitis in rats (Tian et al., 2021). Moreover, these molecules have been demonstrated to inhibit the infiltration of TCD3+ cells and CD68+ macrophages in the intestinal mucosa, thereby alleviating colitis by reducing the production of pro-inflammatory cytokines TNF-α and IL6 by macrophages (Du et al., 2019). The ATF intervention was observed to significantly down-regulate the expression of macrophage markers *F4/80*, *CD68*, *CD11b,* and *CD14*, and to inhibit the expression of *TNF-α*, *IL-1β*, *COX-2*, and *MCP-1*, in a manner consistent with the findings of previous literature reports. These observations suggest that ATF may have the potential to prevent colitis by modulating immune cells and inflammatory factors (Figure 2).

A substantial body of clinical evidence indicates that the intestinal barrier plays a pivotal role in maintaining intestinal health. Macrophages within the lamina propria of the normal intestinal mucosa are devoid of CD14. In the event of LPS-induced apoptosis and necrosis of intestinal epithelial cells, a considerable number of CD14+ monocytes are recruited to the mucosal site (He et al., 2016). Concurrently, LPS is readily absorbed into the bloodstream, where it stimulates monocytes to produce substantial quantities of TNF-α and IL-1β. This, in turn, results in the onset of intestinal inflammation and disruption of the intestinal barrier (Gren and Grip, 2016). The current study demonstrated that DSS intake led to a notable elevation in serum LPS levels and a marked up-regulation in the expression of pro-inflammatory mediators, including TNF-α, IL-1β, and CD14. H&E staining results confirmed that the DSS induced the destruction of intestinal epithelial cells and a substantial infiltration of inflammatory cells. However, these effects were markedly attenuated following ATF intervention, accompanied by a notable elevation in *ZO-1*, *Occludin*, and *Claudin-4* expression (Figure 3). This finding is in accordance with the conclusions of Chen et al., who demonstrated that procyanidins can inhibit the expression of NF-κB and NLRP3 inflammatory vesicle signaling induced by LPS (Chen et al., 2018). This process serves to attenuate intestinal barrier damage in mice with colitis. Furthermore, isoquercitrin (Cibiček et al., 2016), quercetin (Wang et al., 2024), and rutin (Wu et al., 2023) have all been found to safeguard the gut barrier by suppressing the production of pro-inflammatory mediators and by enhancing the release of tight junction proteins and mucins.

TLRs/NF-κB signaling pathway represents a crucial link in the pathogenesis of IBD (Kordjazy et al., 2018). As LPS, a Gram-negative bacterial cell wall component present in the intestinal lumen, crosses the damaged intestinal barrier and enters the intestinal mucosal lamina propria, it is recognized by TLR4. Subsequent binding of LPS to TLR4 activates MyD88 and TRAF6 proteins, initiating downstream signaling and releasing NF-κB dimmers into the nucleus. This process prompts the transcription of a series of inflammation-associated genes, thereby triggering the inflammatory response. In the present study, the increase in LPS content may have contributed to the rise in the expression of its receptor TLR4, while promoting a significant rise in the expression of *NF-κB, MyD88,* and *TRAF6* in colon, implying the activation of the NF-κB signaling pathway. The activation of NF-κB has been observed to upregulate the expression of inflammatory vesicle components, including NLRP3, ASC, and the precursor of Caspase-1 (Yang Q. et al., 2022). Formononetin has been demonstrated to reduce the protein levels of the NLRP3 pathway (NLRP3, ASC, and IL-1β) in the DSS-induced acute colitis mice (Wu et al., 2018). Baicalein has been shown to attenuate TNBS-induced colitis by inhibiting the TLR4/MyD88 signaling cascade and inactivating the NLRP3 inflammasome (Luo et al., 2017). Similarly, we also found that ATF significantly inhibited the DSS-induced activation of NF-κB and NLRP3 inflammasome. From this, we speculate that ATF is able to improve UC by inhibiting the LPS/TLR4/NF-κB/NLRP3 signaling axis.

Flavonoids have been demonstrated to exert a beneficial influence on the regulation of gut microbiota (Shen et al., 2022). Our findings indicate that ATF is capable of effectively reversing the abnormalities of gut microbiota observed in UC mice (Figure 5). The specific differential taxa exhibited varying degrees of correlation with pathological phenotypic indices of UC mice, including the body weight change rate of mice (BWCR), colon length (CL), and disease activity index (DAI). It implied that these differential taxa may play a significant role in the alleviation of UC by ATF. Previous clinical data has shown that the relative abundance of *Escherichia-Shigella* in UC patients was significantly higher than that observed in normal patients (He et al., 2021). *Escherichia-Shigella* is a typical genus of Proteobacteria, and is also a major activator of the LPS/TLR4/NF-κB pathway (Tian et al., 2023). In the present study, the relative abundance of *Escherichia-Shigella* in the cecum contents of UC mice induced by DSS was found to be more than 10-fold higher than that of the control mice. ATF treatment was found to significantly reduce the relative abundance of *Escherichia-Shigella*. The relative abundance of *Escherichia-Shigella* showed a strong negative correlation with the BWCR, and the CL showed a strong negative correlation and a strong positive correlation with DAI. Notably, *Escherichia-Shigella* also exhibited a significant positive correlation with LPS, TRAF, and NLRP3, suggesting that the ameliorative effect of ATF on the DSS-induced colitis may be partly mediated by reducing the relative abundance of *Escherichia-Shigella*.

In addition to *Escherichia-Shigella, Desulfovibrio, Colidextribacter,* and *Oscillibacter* are frequently identified as pathogenic genera in UC. *Desulfovibrio* is capable of producing LPS, and studies have demonstrated that LPS produced by *Desulfovibrio desulfuricans* enhances the transcriptional activity of the NF-κB p65 and IκBα genes in colon intestinal epithelial cells (Kapral et al., 2010). A significant increase in the relative abundance of *Colidextribacter* has been demonstrated to lead to elevated expression levels of NLRP3 inflammasome and a concomitant reduction in the expression of tight junction proteins (Gao et al., 2024). Similarly, *Oscillibacter* may contribute to severe the DSS-induced colitis (Li et al., 2018). The findings of the present study demonstrated that these genera exhibited significant correlations not only with conventional phenotypic indicators of colitis, but also with body inflammation, intestinal barrier function, and the LPS/TLR4/NF-κB/NLRP3 signaling pathway. It was speculated that these genera might also be involved in the process of amelioration of ATF in UC mice.

It has been demonstrated by preceding studies that certain flavonoid monomer components, including phloretin (Wu et al., 2019), rutin (Power et al., 2016), and proanthocyanidin (Sheng et al., 2020), have the capacity to enhance the relative abundance of beneficial bacteria, such as *Bifidobacterium, Akkermansia,* and *unclassified_f__Atopobiaceae,* within the context of the anti-colitis process. The present study found that ATF intervention was also able to significantly increase the relative abundance of these genera, which are associated with the production of short-chain fatty acids. These acids play an important role in maintaining the intestinal barrier (Zeng et al., 2017; Domingo et al., 2008). Meanwhile, *unclassified_f__Atopobiaceae*, *Bifidobacterium*, and *Akkermansia* were found to be significantly and positively correlated with BWCR in UC mice, which was contrary to the findings of DAI. Among them, *unclassified_f__Atopobiaceae* also exhibited a significant negative correlation with several inflammatory factors, including *TLR4*, *NF-κB*, and *NLRP3*. Consequently, *unclassified_f__Atopobiaceae* may be one of the key taxa for ATF in improving UC. Furthermore, in the ATF-treated group, *Akkermansia* was observed to be a highly abundant taxa, although it was not significant in comparison to the DSS group (*p* = 0.0712), *Akkermansia* demonstrated a significant negative correlation with several metrics, particularly the LPS/TLR4/NF-κB/NLRP3 inflammatory signaling pathway. This finding suggests a potential link between *Akkermansia* and ATF-treated UC. In addition to the above taxa, Anaerovoracaceae, *unclassified_f__Anaerovoracaceae*, and *Oscillospiraceae_UCG-005* also exhibited a significant correlation with the UC core indexes. Despite the fact that these taxa have been documented on rare occasions, ATF significantly reversed the aberrant changes of these taxa in UC mice.

In summary, it can be concluded that gut microbiota may play an important role in ATF alleviation of the DSS-induced UC symptoms in mice through modulation of the TLR4/NF-κB/NLRP3 inflammatory signaling pathway, and several classical conditional pathogens (*Escherichia-Shigella*, *Desulfovibrio*, *Colidextribacter* and *Oscillibacter*) and probiotic taxa (*Akkermansia*, *Bifidobacterium* and *unclassified_f__Atopobiaceae*) may be key.

## 5 Conclusion

In this work, high-dose of ATF (100 mg/kg·bw) has been demonstrated to significantly improve symptoms of the DSS-induced colitis. The experiments results showed that ATF may inhibit the occurrence of colitis phenomena, including weight loss, diarrhea, blood in stool, and colon shortening in UC mice by modulating the microbiota-LPS/TLR4/NF-κB/NLRP3 signaling axis. The correlation network between gut microbiota and UC core indicators provides an important basis for elucidating the important mediating role of gut microbiota in ATF alleviation of UC. Several taxa belonging to the classical conditional pathogens (*Escherichia-Shigella*, *Desulfovibrio*, *Colidextribacter* and *Oscillibacter*) and probiotic taxa (*Akkermansia*, *Bifidobacterium* and *unclassified_f__Atopobiaceae*) may be the key mediators. Despite the evidence from animal experiments demonstrating the efficacy of ATF in the treatment of UC, its application in human subjects remains to be elucidated. Furthermore, the types and levels of flavonoids obtained from ATF extracts are subject to variation, thus necessitating further research to identify the key components of ATF’s anti-UC properties. Notwithstanding, this study provides novel insights into the potential of ATF to alleviate intestinal inflammation through dietary intervention, and its multi-targeted characterization of action provides a solid foundation for further research into the development of novel functional foods or plant-derived mucosal repair agents.

## Data Availability

The raw reads of the 16S rRNA gene sequence data were deposited into the NCBI Sequence Read Archive (SRA) database under BioProject accession number PRJNA1204908.

## References

[ref1] Cai R. Yue X. Wang Y. Yang Y. Sun D. Li H.. (2021). Chemistry and bioactivity of plants from the genus Amomum. J. Ethnopharmacol. 281:114563. doi: 10.1016/j.jep.2021.114563, PMID: 34438033

[ref2] Cao S. Y. Ye S. J. Wang W. W. Wang B. Zhang T. Pu Y. Q. (2019). Progress in active compounds effective on ulcerative colitis from Chinese medicines. Chin. J. Nat. Med. 17, 81–102. doi: 10.1016/S1875-5364(19)30012-3, PMID: 30797423

[ref3] Chaen Y. Yamamoto Y. Suzuki T. (2019). Naringenin promotes recovery from colonic damage through suppression of epithelial tumor necrosis factor-α production and induction of M2-type macrophages in colitic mice. Nutr. Res. 64, 82–92. doi: 10.1016/j.nutres.2019.01.004, PMID: 30802726

[ref4] Chen L. You Q. Hu L. Gao J. Meng Q. Liu W.. (2018). The antioxidant procyanidin reduces reactive oxygen species signaling in macrophages and ameliorates experimental colitis in mice. Front. Immunol. 8:1910. doi: 10.3389/fimmu.2017.01910, PMID: 29354126 PMC5760499

[ref5] Cibiček N. Roubalová L. Vrba J. Zatloukalová M. Ehrmann J. Zapletalová J.. (2016). Protective effect of isoquercitrin against acute dextran sulfate sodium-induced rat colitis depends on the severity of tissue damage. Pharmacol. Rep. 68, 1197–1204. doi: 10.1016/j.pharep.2016.07.007, PMID: 27657482

[ref6] Di Tommaso N. Gasbarrini A. Ponziani F. R. (2021). Intestinal barrier in human health and disease. Int. J. Environ. Res. Public Health 18:12836. doi: 10.3390/ijerph182312836, PMID: 34886561 PMC8657205

[ref7] Domingo M. C. Huletsky A. Boissinot M. Bernard K. A. Picard F. J. Bergeron M. G. (2008). *Ruminococcus gauvreauii* sp. nov., a glycopeptide-resistant species isolated from a human faecal specimen. Int. J. Syst. Evol. Microbiol. 58, 1393–1397. doi: 10.1099/ijs.0.65259-0, PMID: 18523184

[ref8] Du Y. Ding H. Vanarsa K. Soomro S. Baig S. Hicks J.. (2019). Low dose epigallocatechin Gallate alleviates experimental colitis by subduing inflammatory cells and cytokines, and improving intestinal permeability. Nutrients 11:1743. doi: 10.3390/nu11081743, PMID: 31362373 PMC6724056

[ref9] Gadaleta R. M. Garcia-Irigoyen O. Moschetta A. (2017). Exploration of inflammatory bowel disease in mice: chemically induced murine models of inflammatory bowel disease (IBD). Curr. Protoc. Mouse Biol. 7, 13–28. doi: 10.1002/cpmo.20, PMID: 28252200

[ref10] Gao Q. Tian W. Yang H. Hu H. Zheng J. Yao X.. (2024). Shen-Ling-Bai-Zhu-san alleviates the imbalance of intestinal homeostasis in dextran sodium sulfate-induced colitis mice by regulating gut microbiota and inhibiting the NLRP3 inflammasome activation. J. Ethnopharmacol. 319:117136. doi: 10.1016/j.jep.2023.117136, PMID: 37704122

[ref11] Gren S. T. Grip O. (2016). Role of monocytes and intestinal macrophages in Crohn's disease and ulcerative colitis. Inflamm. Bowel Dis. 22, 1992–1998. doi: 10.1097/MIB.0000000000000824, PMID: 27243595

[ref12] Håkansson Å. Tormo-Badia N. Baridi A. Xu J. Molin G. Hagslätt M. L.. (2015). Immunological alteration and changes of gut microbiota after dextran sulfate sodium (DSS) administration in mice. Clin. Exp. Med. 15, 107–120. doi: 10.1007/s10238-013-0270-5, PMID: 24414342 PMC4308640

[ref13] He X. X. Li Y. H. Yan P. G. Meng X. C. Chen C. Y. Li K. M.. (2021). Relationship between clinical features and intestinal microbiota in Chinese patients with ulcerative colitis. World J. Gastroenterol. 27, 4722–4737. doi: 10.3748/wjg.v27.i28.4722, PMID: 34366632 PMC8326252

[ref14] He Y. Liu S. Kling D. E. Leone S. Lawlor N. T. Huang Y.. (2016). The human milk oligosaccharide 2′-fucosyllactose modulates CD14 expression in human enterocytes, thereby attenuating LPS-induced inflammation. Gut 65, 33–46. doi: 10.1136/gutjnl-2014-307544, PMID: 25431457

[ref15] Hu Y. Gao X. Zhao Y. Liu S. Luo K. Fu X.. (2023). Flavonoids in Amomum tsaoko Crevost et Lemarie ameliorate Loperamide-induced constipation in mice by regulating gut microbiota and related metabolites. Int. J. Mol. Sci. 24:7191. doi: 10.3390/ijms24087191, PMID: 37108354 PMC10139007

[ref16] Hu Y. F. Zhang X. M. Xu S. Z. Yang S. C. Yang Z. Q. (2018). Analysis of genetic diversity and genetic relationship of Amomum tsao-ko germplasm resources in Yunnan by SSR markers. Chin. Herb Med. 49, 5388–5395. doi: 10.7501/j.issn.0253-2670.2018.22.025

[ref17] Huang Z. Zhao Y. Yang W. Lang L. Sheng J. Tian Y.. (2025). Preparation of flavonoids from *Amomum tsaoko* and evaluation of their antioxidant and α-glucosidase inhibitory activities. Food Chem 25:102177. doi: 10.1016/j.fochx.2025.102177, PMID: 39897968 PMC11786917

[ref18] Kapral M. Węglarz L. Parfiniewicz B. Lodowska J. Jaworska-Kik M. (2010). Quantitative evaluation of transcriptional activation of NF-κB p65 and p50 subunits and IκBα encoding genes in colon cancer cells by *Desulfovibrio desulfuricans* endotoxin. Folia Microbiol. 55, 657–661. doi: 10.1007/s12223-010-0106-6, PMID: 21253915

[ref19] Kim S. G. Becattini S. Moody T. U. Shliaha P. V. Littmann E. R. Seok R.. (2019). Microbiota-derived lantibiotic restores resistance against vancomycin-resistant Enterococcus. Nature 572, 665–669. doi: 10.1038/s41586-019-1501-z, PMID: 31435014 PMC6717508

[ref20] Kim S. E. Kawaguchi K. Hayashi H. Furusho K. Maruyama M. (2019). Remission effects of dietary soybean Isoflavones on DSS-induced murine colitis and an LPS-activated macrophage cell line. Nutrients 11:1746. doi: 10.3390/nu11081746, PMID: 31362418 PMC6723900

[ref21] Klein A. Eliakim R. (2010). Non steroidal anti-inflammatory drugs and inflammatory bowel disease. Pharmaceuticals 3, 1084–1092. doi: 10.3390/ph3041084, PMID: 27713289 PMC4034022

[ref22] Kordjazy N. Haj-Mirzaian A. Haj-Mirzaian A. Rohani M. M. Gelfand E. W. Rezaei N.. (2018). Role of toll-like receptors in inflammatory bowel disease. Pharmacol. Res. 129, 204–215. doi: 10.1016/j.phrs.2017.11.017, PMID: 29155256

[ref23] Li M. Wu Y. Hu Y. Zhao L. Zhang C. (2018). Initial gut microbiota structure affects sensitivity to DSS-induced colitis in a mouse model. Sci. China Life Sci. 61, 762–769. doi: 10.1007/s11427-017-9097-0, PMID: 28842897

[ref24] Liu H. Chen R. Wen S. Li Q. Lai X. Zhang Z.. (2023). Tea (*Camellia sinensis*) ameliorates DSS-induced colitis and liver injury by inhibiting TLR4/NF-κB/NLRP3 inflammasome in mice. Biomed. Pharmacother. 158:114136. doi: 10.1016/j.biopha.2022.114136, PMID: 36535201

[ref25] Luo X. Yu Z. Deng C. Zhang J. Ren G. Sun A.. (2017). Baicalein ameliorates TNBS-induced colitis by suppressing TLR4/MyD88 signaling cascade and NLRP3 inflammasome activation in mice. Sci. Rep. 7:16374. doi: 10.1038/s41598-017-12562-6, PMID: 29180692 PMC5703971

[ref26] Pauly N. J. Michailidis L. Kindred M. G. Flomenhoft D. Lofwall M. R. Walsh S. L.. (2017). Predictors of chronic opioid use in newly diagnosed Crohn's disease. Inflamm. Bowel Dis. 23, 1004–1010. doi: 10.1097/MIB.0000000000001087, PMID: 28368908 PMC5706777

[ref27] Pei L. Y. Ke Y. S. Zhao H. H. Wang L. Jia C. Liu W. Z.. (2019). Role of colonic microbiota in the pathogenesis of ulcerative colitis. BMC Gastroenterol. 19:10. doi: 10.1186/s12876-019-0930-3, PMID: 30642266 PMC6332670

[ref28] Peng L. Gao X. Nie L. Xie J. Dai T. Shi C.. (2020). Astragalin attenuates dextran sulfate sodium (DSS)-induced acute experimental colitis by alleviating gut microbiota Dysbiosis and inhibiting NF-κB activation in mice. Front. Immunol. 11:2058. doi: 10.3389/fimmu.2020.02058, PMID: 33042117 PMC7523281

[ref29] Powell N. Lo J. W. Biancheri P. Vossenkämper A. Pantazi E. Walker A. W.. (2015). Interleukin 6 increases production of cytokines by colonic innate lymphoid cells in mice and patients with chronic intestinal inflammation. Gastroenterology 149, 456–67.e15. doi: 10.1053/j.gastro.2015.04.017, PMID: 25917784 PMC4539618

[ref30] Power K. A. Lu J. T. Monk J. M. Lepp D. Wu W. Zhang C.. (2016). Purified rutin and rutin-rich asparagus attenuates disease severity and tissue damage following dextran sodium sulfate-induced colitis. Mol. Nutr. Food Res. 60, 2396–2412. doi: 10.1002/mnfr.201500890, PMID: 27349947

[ref31] Qu Y. Li X. Xu F. Zhao S. Wu X. Wang Y.. (2021). Kaempferol alleviates murine experimental colitis by restoring gut microbiota and inhibiting the LPS-TLR4-NF-κB Axis. Front. Immunol. 12:679897. doi: 10.3389/fimmu.2021.679897, PMID: 34367139 PMC8339999

[ref32] Shapouri-Moghaddam A. Mohammadian S. Vazini H. Taghadosi M. Esmaeili S. A. Mardani F.. (2018). Macrophage plasticity, polarization, and function in health and disease. J. Cell. Physiol. 233, 6425–6440. doi: 10.1002/jcp.26429, PMID: 29319160 PMC13482560

[ref33] Sharma A. Tirpude N. V. Kumari M. Padwad Y. (2021). Rutin prevents inflammation-associated colon damage via inhibiting the p38/MAPKAPK2 and PI3K/Akt/GSK3β/NF-κB signalling axes and enhancing splenic Tregs in DSS-induced murine chronic colitis. Food Funct. 12, 8492–8506. doi: 10.1039/d1fo01557e, PMID: 34302158

[ref34] Shen N. Wang T. Gan Q. Liu S. Wang L. Jin B. (2022). Plant flavonoids: classification, distribution, biosynthesis, and antioxidant activity. Food Chem. 383:132531. doi: 10.1016/j.foodchem.2022.132531 35413752

[ref35] Sheng K. Zhang G. Sun M. He S. Kong X. Wang J.. (2020). Grape seed proanthocyanidin extract ameliorates dextran sulfate sodium-induced colitis through intestinal barrier improvement, oxidative stress reduction, and inflammatory cytokines and gut microbiota modulation. Food Funct. 11, 7817–7829. doi: 10.1039/d0fo01418d, PMID: 32808642

[ref36] Sun Y. Zhang Z. Zheng C. Q. Sang L. X. (2021). Mucosal lesions of the upper gastrointestinal tract in patients with ulcerative colitis: a review. World J. Gastroenterol. 27, 2963–2978. doi: 10.3748/wjg.v27.i22.2963, PMID: 34168401 PMC8192286

[ref37] Suzuki T. Hara H. (2009). Quercetin enhances intestinal barrier function through the assembly of zonula [corrected] occludens-2, occludin, and claudin-1 and the expression of claudin-4 in Caco-2 cells. J. Nutr 139, 965–974. doi: 10.3945/jn.108.100867, PMID: 19297429

[ref38] Tian G. Wang W. Xia E. Chen W. Zhang S. (2023). Dendrobium officinale alleviates high-fat diet-induced nonalcoholic steatohepatitis by modulating gut microbiota. Front. Cell. Infect. Microbiol. 13:1078447. doi: 10.3389/fcimb.2023.1078447, PMID: 36860985 PMC9968977

[ref39] Tian L. Zhao J. L. Kang J. Q. Guo S. B. Zhang N. Shang L.. (2021). Astragaloside IV alleviates the experimental DSS-induced colitis by remodeling macrophage polarization through STAT signaling. Front. Immunol. 12:740565. doi: 10.3389/fimmu.2021.740565, PMID: 34589089 PMC8473681

[ref40] Wang X. Quan S. Li J. Liu Y. Sun H. Zhang J.. (2022). Protective effects of grape seed proanthocyanidin extract in preventing DSS induced ulcerative colitis based on Pharmacodynamic, pharmacokinetic and tissue distribution. Curr. Drug Metab. 23, 496–505. doi: 10.2174/1389200223666220609151836, PMID: 35692132

[ref41] Wang X. Xie X. Li Y. Xie X. Huang S. Pan S.. (2024). Quercetin ameliorates ulcerative colitis by activating aryl hydrocarbon receptor to improve intestinal barrier integrity. Phytother. Res. 38, 253–264. doi: 10.1002/ptr.8027, PMID: 37873559

[ref42] Whan C. C. Young S. J. Changon S. Su H. S. Eun-Kyung A. Hoon J. Y.. (2018). In vitro anti-inflammatory activity of the components of amomum tsao-ko in murine macrophage raw 264.7 cells. Afr. J. Tradit. Complement. Altern. Med. 15, 26–34. doi: 10.21010/ajtcamv15i2.4

[ref43] Wu M. Li P. An Y. Ren J. Yan D. Cui J.. (2019). Phloretin ameliorates dextran sulfate sodium-induced ulcerative colitis in mice by regulating the gut microbiota. Pharmacol. Res. 150:104489. doi: 10.1016/j.phrs.2019.104489, PMID: 31689519

[ref44] Wu W. Liu L. Zhu Y. Ni J. Lu J. Wang X.. (2023). Zinc-Rutin particles ameliorate DSS-induced acute and chronic colitis via anti-inflammatory and antioxidant protection of the intestinal epithelial barrier. J. Agric. Food Chem. 71, 12715–12729. doi: 10.1021/acs.jafc.3c03195, PMID: 37581468

[ref45] Wu D. Wu K. Zhu Q. Xiao W. Shan Q. Yan Z.. (2018). Formononetin administration ameliorates dextran sulfate sodium-induced acute colitis by inhibiting NLRP3 Inflammasome signaling pathway. Mediat. Inflamm. 2018, 3048532–3048512. doi: 10.1155/2018/3048532, PMID: 29507526 PMC5817291

[ref46] Yang J. Xia X. Du M. Cheng S. Zhu B. Xu X. (2024). Highly effective Nobiletin-MPN in yeast microcapsules for targeted modulation of oxidative stress, NLRP3 Inflammasome activation, and immune responses in ulcerative colitis. J. Agric. Food Chem. 72, 13054–13068. doi: 10.1021/acs.jafc.3c09530, PMID: 38809142

[ref47] Yang S. Xue Y. Chen D. Wang Z. (2022). Amomum tsao-ko Crevost and Lemarié: a comprehensive review on traditional uses, botany, phytochemistry, and pharmacology. Phytochem. Rev. 21, 1487–1521. doi: 10.1007/s11101-021-09793-x, PMID: 35035319 PMC8743105

[ref48] Yang Q. Zhang J. Liu F. Chen H. Zhang W. Yang H.. (2022). *A. caviae* infection triggers IL-1β secretion through activating NLRP3 inflammasome mediated by NF-κB signaling pathway partly in a TLR2 dependent manner. Virulence 13, 1486–1501. doi: 10.1080/21505594.2022.2116169, PMID: 36040120 PMC9450903

[ref49] Zeng H. Huang C. Lin S. Zheng M. Chen C. Zheng B.. (2017). Lotus seed resistant starch regulates gut microbiota and increases short-chain fatty acids production and mineral absorption in mice. J. Agric. Food Chem. 65, 9217–9225. doi: 10.1021/acs.jafc.7b02860, PMID: 28954513

[ref50] Zhang X. F. Tang Y. J. Guan X. X. Lu X. Li J. Chen X. L.. (2022). Flavonoid constituents of Amomum tsao-ko Crevost et Lemarie and their antioxidant and antidiabetic effects in diabetic rats – in vitro and in vivo studies. Food Funct. 13, 437–450. doi: 10.1039/d1fo02974f, PMID: 34918725

[ref51] Zhen Y. Zhang H. (2019). NLRP3 Inflammasome and inflammatory bowel disease. Front. Immunol. 10:276. doi: 10.3389/fimmu.2019.00276, PMID: 30873162 PMC6403142

