# Supplementary material for: Amomum tsaoko flavonoids attenuate ulcerative colitis by inhibiting TLR4/NF-κB/NLRP3 signaling pathway and modulating gut microbiota in mice
Source: Front Microbiol. 2025 Mar 10;16:1557778. doi: 10.3389/fmicb.2025.1557778 (PMC11932354; doi:10.3389/fmicb.2025.1557778)
Supplement: Supplementary file 1 [file Data_Sheet_1.docx]

Supplementary Material

**Supplementary Table 1. DAI scoring criteria for UC mice**

| Feces status | Bloody stools | Weight loss（%） | Score |
| --- | --- | --- | --- |
| Folded, cracked and sausage-shaped. | No colour development within 2 min | ≤1% | 0 |
| Banana-like, soft texture, smooth surface. | 10s latency: light green to green. | 1%＜X≤5% | 1 |
| Soft stools, soft texture, small semi-solid pieces. | The solution turns green, then blue-brown. | 5%＜X≤10% | 2 |
| Mouldable, thin and soft. | The solution turned blue-brown, then darker brown. | 10%＜X≤15% | 3 |
| Slightly shaped and porridge-like. | Reagent adds blue-blackish brown colour. | ＞15% | 4 |

**Supplementary Table 2. Quantitative Real-time PCR reaction program**

| Cycle step | Temperature/℃ | Time/sec | Cycle threshold |
| --- | --- | --- | --- |
| Pre-denaturation | 95 | 30 | 1 |
| Denaturation | 95 | 10 | 40 |
| Annealing and Extension | 60 | 30 |  |
| Melting curve | default settings | default settings | 1 |

**Supplementary Table 3. Primer sequences used for the determination of functional gene expression**

| Primer | Forward sequence | Reverse sequence |
| --- | --- | --- |
| *RPL-19* | GAAGGTCAAAGGGAATGTGTTCA | CCTTGTCTGCCTTCAGCTTGT |
| *TNF-α* | AGACCCTCACACTCAGATCA  TGTTG | TCTTTGAGATCCATGCCGTTG |
| *IL-1β* | TCCATGAGCTTTGTACAAGGA | AGCCCATACTTTAGGAAGACA |
| *IL-10* | AAGGACCAGCTGGACAACAT | TCTCACCCAGGGAATTCAAA |
| *IL-18* | GACTCTTGCGTCAACTTCAAGG | CAGGCTGTCTTTTGTCAACGA |
| *IL-6* | GTTCTCTGGGAAATCGTGGA | TGTACTCCAGGTAGCTA |
| *INF-γ* | ATCTGGAGGAACTGGCAAAA | TTCAAGACTTCAAAGAGTCTGAGGTA |
| *F4/80* | CCCCAGTGTCCTTACAGAGTG | GTGCCCAGAGTGGATGTCT |
| *CD68* | TGTCTGATCTTGCTAGGACCG | GAGAGTAACGGCCTTTTTGTGA |
| *CD11b* | ATGGACGCTGATGGCAATACC | TCCCCATTCACGTCTCCCA |
| *CD14* | CTCTGTCCTTAAAGCGGCTTAC | GTTGCGGAGGTTCAAGATGTT |
| *Ly-6G* | GACTTCCTGCAACACAACTACC | ACAGCATTACCAGTGATCTCAGT |
| *CD115* | TGTCATCGAGCCTAGTGGC | CGGGAGATTCAGGGTCCAAG |
| *Muc-2* | ACGTGTCATATTTGCACCTCT | TCAACATTGAGAGTGCCAACT |
| *ZO-1* | TTTTTGACAGGGGGAGTGG | TGCTGCAGAGGTCAAAGTTCAAG |
| *Occludin* | ATGTCCGGCCGATGCTCTC | TTTGGCTGCTCTTGGGTCTGTAT |
| *Claudin4* | GTCCTGGGAATCTCCTTGGC | TCTGTGCCGTGACGATGTTG |
| *TLR2* | GCAAACGCTGTTCTGCTCAG | AGGCGTCTCCCTCTATTGTATT |
| *TLR4* | AGGCACATGCTCTAGCACTAA | AGGCTCCCCAGTTTAACTCTG |
| *TLR5* | GCAGGATCATGGCATGTCAAC | ATCTGGGTGAGGTTACAGCCT |
| *NF-κB* | ATGGCAGACGATGATCCCTAC | TGTTGACAGTGGTATTTCTGGTG |
| *MyD88* | AGGACAAACGCCGGAACTTTT | GCCGATAGTCTGTCTGTTCTAGT |
| *TRAF6* | ACAGGCCATCCCAAGAATAGG | AAGCCTCTGTTCATACCGTAGTA |
| *NLRP3* | ATTACCCGCCCGAGAAAGG | TCGCAGCAAAGATCCACACAG |
| *Caspase-1* | ACAAGGCACGGGACCTATG | TCCCAGTCAGTCCTGGAAATG |
| *ASC* | CTTGTCAGGGGATGAACTCAAAA | GCCATACGACTCCAGATAGTAGC |


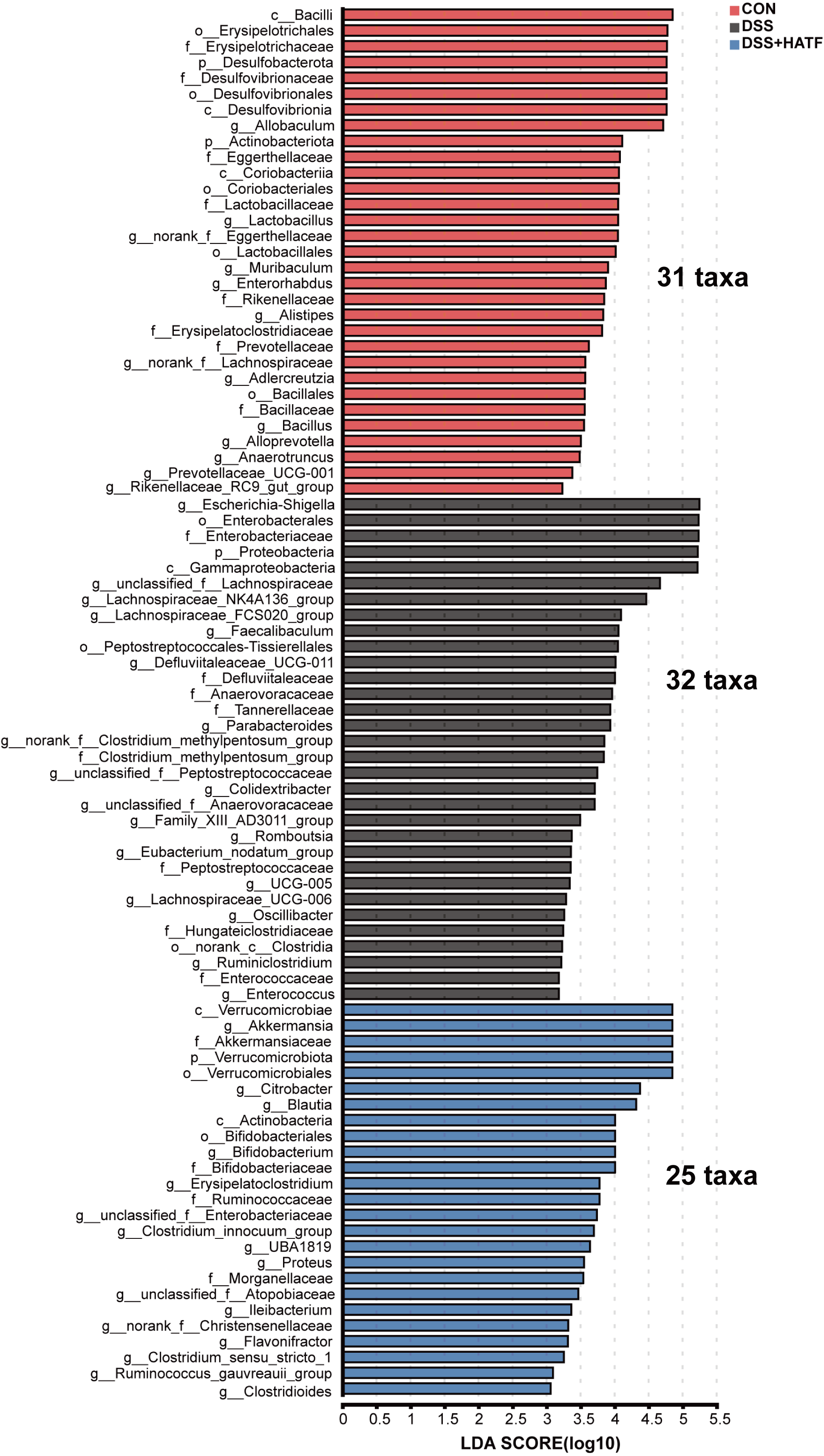


**Supplementary Figure 1. Linear discriminant analysis (LDA)**
